# Identification and description of the sociomotor sub-roles and the Ludogram of Brazilian jiu-jitsu

**DOI:** 10.3389/fspor.2023.1186202

**Published:** 2023-06-14

**Authors:** Vagner Augusto de Oliveira Schmidt, João Francisco Magno Ribas

**Affiliations:** ^1^ Grupo de Estudos Praxiológicos, Centro de Educação, Universidade Federal de Santa Maria, Santa Maria, Rio Grande do Sul, Brasil; ^2^ Grupo de Estudos Praxiológicos, Centro de Educação Física e Desportos, Departamento de Desportos Coletivos, Universidade Federal de Santa Maria, Santa Maria, Rio Grande do Sul, Brasil

**Keywords:** motor praxeology, Brazilian jiu-jitsu, internal logic, sociomotor sub-roles, Ludogram

## Abstract

**Introduction:**

Brazilian jiu-jitsu (BJJ) was conceived to be an oppositional sociomotor practice with an emphasis on self-defense, but throughout the 20th century, BJJ gained sporting features, modifying its internal logic (IL). In BJJ, the richness of the motor itineraries can be revealed in the different sociomotor sub-roles. Considering the absence of research that identifies and describes the sub-roles and the Ludogram of BJJ, the following question was asked: how can the Ludogram of sociomotor sub-roles of Brazilian jiu-jitsu be systematized in accordance with its internal logic?

**Methods:**

This work is characterized as theoretical research that is dedicated to rebuilding theories and concepts with a view, in immediate terms, to improving theoretical foundations. In this study, a theoretical reconstruction of BJJ’s operating dynamics was carried out, identifying roles and sub-roles, culminating in the construction of a Ludogram. The praxeological analysis was divided in two stages: (1) Description of the BJJ sub-roles based on sports rules and video analysis; (2) Systematization of the BJJ Ludogram. Eight public videos with unrestricted access were selected of fights from the 2018 BJJ World Championship. The sample was considered based on the following criteria: convenience, typicality, and saturation.

**Results and Discussion:**

The 26 identified and described sub-roles of BJJ indicate the richness of choices and possible paths to be followed by fighters within this itinerary of motor interaction. These different BJJ sub-roles described in this research highlight the importance of the concept of praxis communication, specifically, motor counter-communication, since many of the dynamics between a fighter’s subroles refer to the choices that the opponent indicates for the motor dialogue. BJJ requires from fighters incessant activations on aspects related to sociomotor intelligence, such as the need for sociomotor empathy, motor strategy, to anticipate anticipations, pre-acting, developing the capacity to make motor decisions, to recognize the affective, cognitive, relational, and organic loads activated during the fight, and to develop their motor conduct. In this sense, the Ludogram was elaborated, which enables future praxeological analyses of the sub-roles and motor conducts of any subject who wants to assume the sociomotor role of a BJJ fighter according to the rules of this Brazilian combat sport.

## 1. Introduction

The theme of internal logic (IL) from motor praxeology (MP) refers to a process of dense, systematic, and scientific description of elements that reveal the central/nuclear, structuring, and systemic aspects of a motor practice. MP has often been compared with music theory, in particular, with the musical notation process that consists of structuring/systematizing a musical work into scores. But the score of a song does not manifest itself. It needs a protagonist, the performer, the musician, to bring it to life. And a good musician goes back and forth from the score to interpretation and from interpretation to the score as many times as necessary to reach a synthesis of that work.

In the field of physical education, before Parlebas' proposal, there was no possibility of basing its pedagogical practice on a grammar of motor practices. The pedagogical practice, which is dedicated to the teaching-learning process of sports games, as well as the reflection on the same, was, and still is, guided by apparent elements of motor practices, with pedagogical objectives that do not value the subject who moves inside the game. The emphasis is only on movement, with techniques isolated from the game system, without understanding the meaning of such actions, whether directly related to internal logic or related to aspects of external logic (such as history, social context, pedagogical objectives, culture).

Concerning the aspects of the external logic of BJJ, according to Gracie (1), historically it is recognized that forms of jiu-jitsu were already practiced in the mountains of India for at least 2,500 years, later spreading through China about 400 years ago, and further strengthened as a fighting art in Japan. Only in the 20th century did Japanese jiu-jitsu culture reach South America. According to Lise and Capraro (2), the arrival of jiu-jitsu in Brazil was initially due to the arrival of two Japanese fighters, Sada Miyako and M. Kakiora, whose role was to teach the techniques of this martial art to Brazilian sailors in 1908 even before it became known to the Brazilian population. It should be noted that this version is opposed to the hegemonic story reported by Gracie (3), which argues that Mitsuyo Esai Maeda, also known as “Konde Koma”, would be responsible for the arrival of jiu-jitsu in Brazil, in Belém do Pará in 1915. According to Lise and Capraro (2), Miyako's arrival in Brazil occurred approximately six years before the arrival of Konde Koma, who would have landed in Brazil in 1914 and not in 1915 as mentioned (3).

However, the history of this combat sport in Brazil is sometimes confused with the history of the Gracie family. According to Gracie's historical version, Gastão Gracie became a jiu-jitsu enthusiast and took his eldest son, Carlos, to learn from Konde Koma. From that moment on, a whole dynasty of brothers, grandchildren, and great-grandchildren would practice and develop this combat sport, spreading it throughout Brazil and, more recently, to the world (3). In the 20th century, the jiu-jitsu practiced by the Gracie family began to present ground submission techniques that were more sophisticated than traditional Japanese jiu-jitsu (1). Changes and technical adaptations related to practice have altered the complexity of jiu-jitsu principles, making it even more efficient as a self-defense system (4). In this sense, according to Lise and Capraro (2), the importance of Konde Koma in the process of disseminating jiu-jitsu in Brazil is undeniable, as is the importance of the surname Gracie for the worldwide recognition of jiu-jitsu. In addition to the emphasis on self-defense, BJJ is currently organized as a sport, with the International Brazilian Jiu-Jitsu Federation (IBJJF) as the main institution that organizes the rules and championships of this Brazilian fighting modality worldwide.

Regarding the general aspects of the internal logic of BJJ, a preliminary study carried out by Schmidt and Ribas (5) revealed that, according to praxeological analyses, this corporal fighting style, conceived as a praxeological system, is characterized as: a sociomotor practice of opposition; unrolled in a stable environment; a duel between two individuals situated at almost null guard distance, with motor interactions directed at the opponent's body (joint locks, strangulations, falls, projections, imbalances, immobilizations, etc.); in addition, both participants assume the same sociomotor role, that is, they have the same rights, obligations, and restrictions regarding motor interactions.

BJJ has direct praxis communication of motive counter-communication and indirect praxis communication, through praxemas and gestures. But, due to the fact that the responsibility for victory or defeat always falls to one of the participating fighters, BJJ has been mistakenly conceived as a motor practice of individual action. However, characterizing it as a psychomotor or individual action sport refers to organizing didactic situations in an internal logic different from their needs for direct and indirect practical communication (5). The authors conclude that motor counter-communication interactions require participants to develop the competence to read and interpret the opponent's body messages. At the same time, fighters must make their interpretations difficult, seeking to confuse their real intentions so that they are not decoded by the opponent, which determines complex motor behaviors of strategy and motor decision.

The characterization of these aspects of IL becomes essential to guide the teacher-coach's pedagogical practice. Ribas (6) pointed out that this new knowledge contributes to a better understanding of the elements of motor practice, in addition to teaching with more consistency and meaning. The aforementioned author also pointed out that the concepts of IL must be shared, constructed, and appropriated by student-athletes as well. Parlebas (7) added that the objectives and pedagogical effects of a pedagogical practice are closely related to the IL of the practice, as they refer, at a first level, to the very improvement of the motor practice by the learning subjects, as well as to the development of the participant's personality through their motor behavior.

Still in relation to the pedagogical aspects, it is highlighted that the IL should not be a determinant of the motor behavior; it is not a path that goes only in this direction. It is understood that this relationship must work in both directions, and may even cause the pedagogy of motor behaviors to transform elements of IL. Parlebas (7) reinforced this relationship in the following statement: “… *the internal logic of a motor practice can be reinterpreted from the outside, by an “external” logic that attributes new and unusual symbolic meanings to it* (p. 307)”.

In the case of BJJ, IL should not be placed on a pedestal as the ultimate goal. Along the way, it is possible that other possibilities for practicing Brazilian jiu-jitsu will arise, with simpler, safer rules and, above all, adequate to the pedagogical needs of the teacher-coach. That is, a permanent Üycle of consideration of the internal logic and its relationship with the motor behavior and returning to the pedagogical practice and its objectives.

Considering the pedagogical aspects, Schmidt and Ribas (5) defended the need for further praxeological studies of BJJ to unveil its praxeological system. The production of this scientific knowledge is sought to overcome the superficial understanding regarding the structures and dynamics of motor practices. MP instrumentalizes scientists and teachers to develop their studies and pedagogical practices in order to understand the internal logic relationships—motor conducts.

In practices with only one sociomotor role, as in the case of BJJ, the richness of the motor itineraries which the participating fighters can go through will appear at a second level of analysis, in the socio-motor sub-roles, which, according to Parlebas (7), are dependent on the sociomotor status and its dynamic translation of sociomotor roles. A sociomotor sub-role is defined as the “…ludomotor sequence of a player considered as the basic behavioral unit of the strategic functioning of a sports game.” (7).

Sub-roles refers to a type of motor behavior that groups actions considered strategically equivalent. Parlebas (7) elucidated by exemplifying that “the multiple ways that a player has to pass the ball to a teammate (with one hand, with both hands, standing, running, with the arm bent, turning around, etc.) will be grouped in the same sub-role that we label as ‘pass’.” Parlebas (8) demonstrated that in the case of field football, the role of the field player can be subdivided into sub-roles such as: passer, finisher, dribbler, recoverer, etc.

The catalog of sociomotor sub-roles of a sports game is not enough to know its functioning mechanisms. One must discover the syntaxes that combine these units, that is, the network of possible linkages, dynamics, exchanges, and inversions of sub-roles. A succession of sub-roles constitutes a summary of a player's motor behavior when expressing his choices, preferences, and motor decisions, that is, aspects related to motor behavior and its intimate relationship with the internal logic of the sports game (7).

Sociomotor sub-roles represent the matrices in which all the potential sequences that can be updated by the players are pre-programmed, invariant networks that authorize an infinity of variations, a trajectory that can be drawn in the form of a Ludogram (7). According to Parlebas (7) the Ludogram is the “… graphical representation of the sequence of the sociomotor sub-roles (and possibly of the sociomotor roles) assumed by a player successively during the development of a sports game”. the Ludogram is an instrument for studies referring to the player's motor strategies, as well as to understand the relational, motor decisions, and organic and affective aspects of a participant, that is, his motor behavior (7).

Thus, with the aim of creating scientific parameters for combined analyses of the internal logic-motor conduct relationship of BJJ, the research problem arises: how can the Ludogram of sociomotor sub-roles of Brazilian jiu-jitsu be systematized in accordance with its internal logic?

## 2. Materials and methods

This work is characterized as theoretical research that, according to Demo (9), is “*dedicated to reconstructing theories, concepts, ideas, ideologies, polemics, with a view, in immediate terms, to improving theoretical foundations*”. In this study, a theoretical reconstruction of BJJ's operating dynamics was carried out, identifying roles and sub-roles, culminating in the construction of a Ludogram, a process called praxeological analysis.

As for the scope of the research (level of explanation), the study is characterized as being exploratory and descriptive. Exploratory studies serve to make a relatively unknown phenomena familiar; to obtain information about the possibility of carrying out a more complete search related to a particular context; research new problems; identify promising concepts or variables; and establish priorities for future research or suggest assertions and postulates. Regarding the descriptive scope, the objective was to describe phenomena, situations, contexts, and events; that is, detailing how they are and how they manifest themselves, seeking to specify the properties, characteristics, and profiles of a phenomenon that is submitted to analysis (10). In this research, such a description refers to a praxeological analysis that consists of articulating the concepts and tools of MP with the aim of revealing the internal logic of BJJ to structure and systematize the BJJ Ludogram.

With regard to the praxeological analysis, BJJ's operating logic was described from the relationship between: MP concepts and tools; the official rules of the modality found on the website www.cbjj.com.br; and the observation of motor behaviors through a non-participant systematic observational analysis of BJJ fight videos. This procedure made it possible to map the motor situations of BJJ to facilitate its understanding, a technique known as modeling (11). From this perspective, reality is represented in the form of models of praxeological analysis and follows some dimensions of interpretations and different methodological guidelines including, among these elements, the universals of sports and games (7).

### 2.1. Methodological strategy for analyzing BJJ's internal logic

The tools and concepts of MP were the instruments used to interpret the characteristics of the internal logic. For the praxeological analysis of the internal logic of BJJ, five of the seven universals of sports and games were used, which represent the basic operating structures of the internal logic of motor practices (7). This article presents, specifically, the results of the identification and description of the BJJ sociomotor sub-roles with the aim of systematizing the BJJ Ludogram.

In addition to the information present in the IBJJF rulebook, it was necessary to complement this analysis by observing the motor behavior of fighters who participate in international competitions. According to Parlebas (7, 8), in order to characterize the sociomotor sub-roles, it is necessary to know the rules of the game and take into account its norms, as well as to carefully observe the behaviors developed in the practice space. The observation of motor behaviors was carried out through a systematic, non-participant, observational analysis of videos of BJJ fights, public videos from youtube.com from the IBJJF channel with unrestricted access. The observational analysis of the videos of the fights had the function of contributing to:

• Identification of the sociomotor sub-roles;
• Description of the classes of motor interactions of each sub-role;
• Systematization of the Ludogram.

The five universals of games and sports were articulated with the concepts of the theory of motor action, mainly with the terms indicated at the end of each of the universals present in the Lexicon of Motor Praxeology, which, according to Parlebas (7), are interrelated. This articulation allowed a deductive analysis based on BJJ rules and the observational analysis of the operating models that represent the basic operating structures of the internal logic of BJJ. This methodological systematization is demonstrated in Table 1, in which the universal “the graph of changes in sub-roles” stands out for this article.

**Table 1:** Methodological scheme for analyzing the internal logic.

**The Game: Universals of games and sports**					
Universals of games and sports	The network of motor interaction	The network of score interaction	The score system	Sociomotor role exchange network	The graph of changes in sub-roles
Concepts and praxeological tools used to describe the internal logic	Motor communication; Motor counter communication; Score interaction; Duel; Graphic	Motor communication; Motor counter communication; Score interaction; Duel; Graphic	Score; Support; Universal; Punctuation; Score interaction	Sociomotor status; Sociomotor role; Universal; Graphic; Internal logic; Sports space; Categories	Sociomotor sub-role; Ludogram; paper network; sociomotor role; Graphic
Search sources	IBJJF Rule book				IBJJF rulebook + BJJ fight videos

### 2.2. Video sample selection

The criteria for choosing the sample of videos included convenience, typicality, and saturation. Convenience, due to the possibility of analyzing the videos of fights from one of the main BJJ events available on the internet with public and unrestricted access: fights from the 2018 Brazilian Jiu-Jitsu World Championship with reference fighters in the modality. Typicality, because, according to Lakatos and Marconi (12), in certain cases, when it is not possible to make a probabilistic sample, sampling by typicality tries to seek a representative sample by other means. One way to do this is to look for a subgroup that is typical of the population as a whole. In this case, it is typical because the internal logic pre-orients the elementary actions of anyone who submits to its rules, which is the specific case of these fights.

Considering that the specific objective is to reveal the representative sociomotor sub-roles of the fight, the total number of samples to be analyzed in relation to the selected videos was not initially defined. Therefore, the sample saturation technique served as a reference to define, during the research, when to interrupt such analyses. From the fifth video, the identification of new BJJ sub-roles was significantly reduced, with this reaching saturation once the eighth video was analyzed. It was decided to carry out the descriptions of the sub-roles of the fighters who participated in the 2018 world championship organized by the IBJJF, that is, who fight by the same rules that are serving to characterize the internal logic of this work.

According to Parlebas (7), for a system of rules given, the number of sociomotor roles is constant, but the same does not occur with the sub-roles, which can vary according to the characteristics of the players, their age, capacity for initiative, and technical and tactical levels. These variables will modify the range of assumed sub-roles. From this perspective, we sought to analyze the motor behaviors of high-level athletes in order to try to identify and recognize as many sub-roles as possible, considering their initiative capabilities and technical and tactical levels. However, the author explains that the sub-roles leave some margin, as they depend on the fighters' decisions to assume them. Therefore, future research may corroborate or advance what has been produced so far regarding the identification and description of BJJ's sociomotor sub-roles.

For reasons of standardization, the videos chosen for analysis were from the black belt category, from the final and semifinal fights of the male and female categories of the same championship, available on the IBJJF channel on the website youtube.com, consulted during the second half of 2019. It was decided not to define a weight category so that the possibilities of minimum strategic actions were as wide as possible, in order to avoid some sub-roles not being characterized, affecting the identification and description of the sub-roles. Therefore, Table 2 presents the criteria used to choose the analyzed videos:

**Table 2:** Criteria for selection of fight video samples.

**Criteria for selection of fight video samples**
• Videos published by the IBJJF channel on youtube.com.
• Videos from the “World championship 2018” playlist.
• From the videos in the “World Championship 2018” playlist, the videos that present the following in the video description will be included: the weight category, belt category of the Úthletes, and phase of the championship that the fight is occurring.
• The fights must be from the finals and semifinals of the 2018 World Championship, male and female black belts, and any weight category.
• Full fight videos (which show the fight from start to finish) and which show the time and score in the fight video.
• Total quantity of videos on the World Championship 2018 Brazilian Jiu-Jitsu playlist: 29 videos. • Total analyzed videos: 8 videos.

The sociomotor sub-roles were identified and described over the time of the video of each fight according to the spreadsheet built specifically for this observational analysis, considering the criteria described by the motor action theory. Based on Parlebas (7, 8, 13, 14), Table 3 shows the concepts that were used as criteria to identify and describe the sociomotor sub-roles of BJJ.

**Table 3:** Synthesis of concepts used to identify and describe BJJ's sociomotor sub-roles.

Concepts used as criteria for identifying and description BJJ's sociomotor sub-roles
• Sub-role is the ludomotor sequence of a player considered as a basic behavioral unit for the operation of a sports game.
• It is necessary to know the rules of the game and take into account its norms, as well as carefully observe the behaviors developed on the ground.
• Some minimum strategic units can last for a few minutes and others a few seconds;
• Sub-role refers to types of motor behavior that group actions judged to be equivalent from a strategic point of view.
• The sub-roles will correspond to interactions that lead fighters to take initiatives and motor decisions (which may succeed or fail).
• A sub-role has its own unity both within the player's internal logic and strategic logic.
• A sub-role is constituted as a praxis sequence that can be considered as a minimum unit of tactical interaction of the operative functioning of the sports game.
• The elementary strategic unit is undoubtedly the sub-role, that is, it is what gives meaning to the partial elements whose relationship allows its definition. Every partial element and every indicator can be read as a clue.

In order to carry out the observations of the videos, spreadsheets were created in which it was possible to describe the motor behaviors and identify the sociomotor sub-roles of BJJ. The spreadsheet also presented general information about the fight (name of the fighters, stage of the fight, championship, and video link), as shown in Table 4.

**Table 4:** Identification of BJJ sub-roles (model).

Praxeological analysis worksheet of sociomotor sub-rolesidentification and description of the sub-roles of Brazilian jiu-jitsu fighters					Page
Fight:	Stage of fight:		Championship:		
Video link:					
Video time	White Kimono Fighter (WKF)	Blue Kimono Fighter (BKF)	Strategic role		Comments
			WKF	BKF	
	Sub-roles	Sub-roles			
					
					
					
					

From the results obtained in the spreadsheet and the deductions made according to the rules of practice, a table was built identifying and characterizing the sociomotor sub-roles with the description of the corresponding motor interaction classes. The results of the study will be presented below.

## 3. Results and discussion

### 3.1. Internal logic of Brazilian jiu-jitsu: the sociomotor sub-roles

The identification of the sociomotor sub-roles highlights the structural part that is closest to the interactions of BJJ fighters, while it makes it possible to understand the motor conduct of the participants when unveiling their initiatives and practical choices. Therefore, it allows a combined analysis of the game's objective logic and the player's subjective conduct (7). Lagardera and Lavega (15) contributed by indicating that each sub-role represents a strategic action whose dimension, being basic or minimal, allows a very precise approximation to what happens on the field of play.

Since BJJ only has a sociomotor role, it could indicate that it would not activate different possibilities regarding the motor conducts of its participants. But, in BJJ, this sociomotor role allows the fighter to assume different minimum units of opposition interaction, revealing a potential for activating and developing the motor conducts of the participating fighters.

According to Parlebas (7), the concept of sociomotor sub-roles can illuminate the two inseparable aspects of motor action. On the one hand, it highlights the objective system of essential strategic units necessarily carried out by any player, however original they may be; on the other hand, it manifests the praxis choices made by the individual, indicates the sequences preferred by any participant, and reveals the forms of relational expression proper to each one.

Parlebas (7) points out that every sociomotor sub-role must be labeled by the researcher with a noun that highlights the dominant interaction linked to the observed sequence, which represents an elementary action that must be indicated by a verb. Remember that, in the case of the sociomotor role, it is impossible to have the same clarity because it encompasses a set of different actions. What should be done is to identify the sociomotor role through a label with a broader—and, therefore, more blurred—meaning, which will have a denominative value instead of a descriptive one. In this way, the sociomotor role of BJJ was denominated as “Fighter of Brazilian Jiu-Jitsu”. Table 5 below shows the identification and description of the different sociomotor sub-roles of BJJ. As it is a Brazilian combat sport, the names of the sub-roles are also presented in Portuguese.

**Table 5:** Identification and description of sociomotor sub-roles in Brazilian jiu-jitsu.

Sociomotor Role: Brazilian jiu-jitsu fighter	
Sociomotor sub-role corresponding motor interaction classes	
ON ALERT *(em alerta)*	This sub-role implies a predisposed, alert, attentive attitude on the part of the fighter, ready to intervene at any moment with the opponent, before the search for a grip on the kimono or other contact with the opponent (body) that aims at some control of fists, hands, neck, etc. The fighter, usually, is in the initial moments of the fight, standing up or after a dispute for a position in which both do not have a grip on the opponent's body or kimono. The fighter is in this sub-role until they seek another driving interaction with the opponent.
GRABBER*(agarrador)*	The fighter tries to interact with the opponent in search of a grip on the kimono or an initial grip on the opponent's body in a way that allows initial control. When the fighter tries to grip with no definition of which motor interaction will occur next. The fighter seeks to gain initial control over the opponent through the grip on the opponent's kimono or a grip on the opponent's body. When the fighter tries to grab to control the hands, wrists, ankles, shins, knees, etc., without subsequent direct interaction. This motor interaction was identified as a sub-role, as it is not possible to deduce another strategic intention of such a grip through the observational analysis of its motor behavior, since in all of them the grip breaks down. It remains in this sub-role until it finally changes its motor behavior. Contrary to this, there are cases in which the fighter takes hold of the kimono and immediately tries to pull the opponent to their guard or even grabs the kimono to try to take down or to try to dominate the back. In these cases, each of these interactions is considered as a separate sub-role, in which the grab of the kimono is a partial element, that is, it is part of a dominant interaction, with another strategic objective, another sub-role. In BJJ, grabbing the opponent's kimono is part of many driving interactions. But in the case of the sub-role that was called “grabber”, specifically, what can be observed is the search for the grip on the opponent's kimono or body for an initial domain; the fighter can also be observed changing grips from one collar to another, or from one sleeve to another, or from one pantleg to another, usually with both fighters standing. Whenever this grappling interaction does not evolve immediately into a possible motive interaction of taking down, grabbing the opponent's back, pulling guard, etc., it is characterized as the grabber sub-role.
DODGER*(esquivador)*	The fighter unfolds their body with the intention of not being grabbed by the opponent who seeks to control their kimono or have some control over their fists, hands, arms, or neck. They unfold, and dodge being grabbed by the opponent. Movements of the arms, hands, and legs can be used to avoid the opponent's grip.
FINISHER*(finalizador)*	The fighter seeks to apply a driving interaction that objectively can lead their opponent to submission or giving up (chokes, keys on joints and bones). Attempts to finish the fight before the expected regular time of the fight. If they succeed in submission, the duel ends, declaring them the winner.
SUBMISSION DEFENDER*(defensor de finalização)*	The fighter who suffers a finishing motor interaction, tries to defend themselves preventing the opponent from succeeding in submission, explicitly seeks to escape or defend themselves from the blow. If the fighter is not clearly defending themselves from the blow, they should not be considered as in this sub-role. Giving up gesture: the fighter taps twice with the palm of the hand on the opponent, on the ground, or on themselves, manifestly and visibly giving up the fight; or when the athlete taps their feet twice on the ground when their arms are trapped by the opponent. If they fail to defend themselves from submission and issue a gesture of withdrawal or even a cry of pain, the combat ends, declaring them as defeated.
GRAPPLE BREAKER *(quebrador de pegada)*	This sub-role involves trying to rip off or break the grips the opponent makes on their kimono or some part of their body, seeking to break the grip dominance that the opponent maintains. This sub-role should not be confused with interactional situations in which breaking the grip is part of a sub-role, such as, for example, breaking the grip to try to pass guard, or to attempt a takedown. In this sub-role, strategic intent is restricted to breaking the hold caused by the opponent's grip.
THROWER*(quedador)*	Starting from an initial movement with both feet on the ground, this sub-role seeks to throw the opponent to the ground. This sub-role can be scored (two points) when: “one of the athletes, starting with an initial movement with both feet on the ground, throws the opponent to the ground on their back, sideways, or makes them fall sitting down, keeping the fight on the ground, and stabilizing the position on top for three seconds” (16).
THROW DEFENDER*(defensor de queda)*	The fighter, in a standing fight, tries to balance themselves and lock their body, stabilizing their body in a standing position, in order to avoid being thrown to the ground. Alternatively, when already thrown to the ground, they try to get up again, trying to stay on their feet, avoiding as much as possible attempts by their opponent to control the fight on the ground and receive points for the interaction of taking down, for example.
GUARD PULLER*(puxador de guarda)*	This sub-role intends to pull the opponent into their leg guard in a ground fight. It could be a transitional interaction from standing to ground fighting. Or every time both are already engaged in the fight on the ground and the opponent who is usually on top moves away, causing the fighter to try to grab to get closer to the opponent, pulling them back to their leg guard; after this interaction, the sub-role changes. The fighter in the fight on the ground and underneath seeks to grab the opponent who moves away from their control of the legs to have the opponent under their guard again.
SWEEPER*(Raspador)*	This sub-role can be scored (two points) when: the fighter starting from a leg guard position (closed guard, half guard, open guard) manages to reverse the position, forcing the opponent who was on top to stay on the bottom in the fight on the ground and manages to stabilize that position for three seconds to earn the score (16). A good indication of the beginning of this interaction can be seen every time the sweep manages to unbalance their opponent with the intention of sweeping/reversing the opponent, causing their opponent to manifest a visible motor behavior in defense of the sweep, that is, they force the opponent who is on top stuck in the guard changing their leg positions to rebalance themselves, opening their base with their legs, for example, or fall with the body sideways, trying to defend and come back, not accepting the sweep/inversion by the sweeper. At the end, it is necessary to analyze which driving interaction will occur, always considering the guard relationship between the opponents to define which sub-role will be assumed. If, at the end of the inversion, the opponent turns their back up, on all four supports, and the athlete who started the inversion controls the opponent's back without the need to place the hooks, but keeps the opponent with at least one knee still on the ground, thus it is configured as the end of this interaction. If, during the inversion, the opponent stands up, but the sweeper maintains control of the necessary grips to continue the sweep even if they need to stand up and take the opponent down, it is also considered as a sweeper sub-role. It is noticed that even having used a takedown, its tactical intention refers to the interaction of a sweep, according to the rules of the modality. When both fighters are sitting down doing 50/50 guard (both fighters sitting on the ground with one of their legs intercrossed with the opponent's), the one who tries to get up and stay on top will also be considered as a sweep interaction.
GUARD CONTROLLER*(controlador de guarda)*	In a ground fight, the fighter who is on the bottom with the opponent in guard or half-guard or open leg guard tries to prevent the opponent who is on top from crossing their leg guard. The fighter seeks to keep the opponent under their leg guard control and does not demonstrate behavior with the objective of sweeping the opponent according to the sweeper sub-role. This sub-role sometimes presents itself as a behavior of little movement, just control/offside, especially with the legs, on the opponent who is trapped in its guard. Hand grips on the opponent are crucial for this domain, but the dominant interaction comes from controlling the opponent's legs, preventing them from passing their leg guard and interacting directly with their torso.
GUARD PASSER*(passador de guarda)*	The fighter seeks to break through the opponent's leg guard, overcome their guard, and cross the opponent's legs that control them, with the intention of seeking some free domain of the opponent's leg guard; this demonstrates motor behavior that they really want to cross or pass the opponent's legs in search of approaching the opponent's trunk without direct interaction with their legs. This sub-role is subject to scoring (three points): whenever the fighter manages to overcome the opponent's leg guard, controlling the opponent's guard-free trunk and stabilizing in that position for three seconds, the score will be marked.
SWEEP DEFENDER*(defensor de raspagem)*	The fighter demonstrates imbalance and seeks to rebalance, defend, and prevent the sweeping interaction performed by the opponent who is below. Or when both fighters are sitting down doing 50/50 guard, the one who tries to prevent the opponent from going up gets up and stays on top, avoiding the sweep interaction; in this case, it should be considered as in the sweep defender sub-role.
TRUNK CONTROLLER*(controlador do tronco)*	A fighter free from the opponent's leg guard seeks to maintain control of the opponent's torso on the ground; this control can be transverse or longitudinal in relation to the opponent's body. It is trunk control that does not refer to sitting/mounting the opponent's trunk from the front or back, nor to dominating the opponent's back, nor to controlling the trunk using the knee on the belly. This description point deserves an important consideration regarding this sub-role and others referring to different domains on the opponent's torso that the fighter seeks throughout the BJJ fight: whether to subdivide the different domains of the opponent's trunk or not. Would they all be the same sub-role (trunk controller)? After examining in detail the internal logic of BJJ, through its rules and the influence of the scoring system, it was understood that one cannot consider all domains on the trunk as the same sub-role, since one of the characteristics pertinent to the concept of sub-role says respect, first, the influences that lead fighters to take initiatives and motor decisions (which may succeed or fail); second, to characterize a sub-role, it must be considered that it has its own unity both within the internal logic and the strategic logic of the fighter. Therefore, in this case, both definitions of the motor action theory induce the researcher to deduce that it is necessary to subdivide the different domains of the trunk. The scoring system encourages/channels the fighter to make different motor decisions based on this interaction. From this torso control interaction, the fighter will be able to try to sit/mount on the torso from the front, from the back, put the knee on the belly, dominate the opponent from the back, or force the opponent to submit. The interaction itself is already different (sitting down, placing a knee, controlling the back), as such interactions are worth points, leading the fighter to take initiative and make motor decisions. In addition, the rule punishes the fighter who is controlling the opponent's torso in this situation for more than 20 s, forcing them to look for other sub-roles. This understanding of the sub-role concept that leads the fighter to make different motor decisions and seek other domains will also weigh in the characterization sequence to define the different escapes of each of these domains, since the fighter who suffers the interaction of each one of these sub-roles should not accept such an interaction, as it will reflect on the score, leading them to decide strategically and seek initiative again by not accepting, therefore, that the different domain interactions take place.
FRONT TRUNK MOUNTER*(montador do tronco de frente)*	The fighter who tries to sit on the trunk of the opponent who is facing them. The criterion for dividing the sub-roles took into account the scoring system, as it leads the fighter to make decisions. The rules allow points to be accumulated by going from a front mount directly to a back mount. This sub-role can be scored (four points) when: the athlete who is on top and already free of the leg guard sits on the opponent's torso facing forward and keeps both knees or one foot and one knee on the ground, facing the opponent's head and with up to one arm of the opponent trapped under their legs, keeping it that way for three seconds (16).
BACK DOMINATOR *(dominador das costas)*	The fighter who seeks to dominate the opponent's back (control/immobilize the opponent from the back). Situations in which the fighter already has their opponent on their back and seeks to evolve to control and dominate their opponent even more through grips and dominance of the back. It appears in all situations in which the fighter is faced with the opponent's back to them at the same time that they seek to evolve in that domain of the opponent's back. This is a scoring sub-role (four points) when: the athlete dominates the opponent's back, placing the heels on the inner part of the opponent's thighs, without crossing the feet, and being able to imprison even one of the opponent's arms without the leg that imprisons the arm passing the shoulder line, and keeping it under control for three seconds (16).
GUARD RECOVERY PREVENTER *(impedidor da reposição de guarda)*	The fighter, after losing some free control of the opponent's leg guard (back control, front and back mount, placing knee on belly, lateral trunk control), demonstrates motor behavior of not accepting/preventing the opponent's guard replacement, defends the replacement to stay free of the guard. It is not possible to identify which domain will do, what is predominantly observed is the attempt to prevent the replacement of guard that the opponent tries to do. An important detail: in the guard-passing sub-role, a similar situation happens; however, the dominant interaction is passing the guard and then seeking some control. The final phase of the pass also appears something like preventing the replacement, but in the case of the pass it is the final phase of the interaction, that is, they are still in the sub-role of passing guard until there is some dominance over the opponent free of guard. Therefore, the replacement impeding sub-role is a transitional position of the fighter who had control over the torso but the opponent has already defended themselves against these controls, freeing themselves to try to replace the guard. The fighter who prevents the replacement, tries to look for some free domain of the guard, when they demonstrate observable behavior of one of these domains, they change the sub-role.
ESCAPER FROM THE DOMAIN OF THE BACK*(escapador do domínio das costas)*	The fighter tries to avoid, to defend, or to get out of the opponent's back grip (remove the hooks, rotate the torso, escape/displace the hip). This sometimes presents itself under strong immobilization or little movement.
FRONT-MOUNTED ESCAPER*(escapador da montada de frente)*	The fighter, in an attempt to prevent the opponent from mounting their trunk from the front, or when already mounted, defends themselves by pushing the opponent's legs, unbalancing the opponent in an attempt to escape. This sub-role sometimes uses hip strength, pushing the opponent upwards, trying to move away from the opponent to find some space. The fighter looks for some grip on the opponent's body that gives them the minimal control to perform an exit from that control. This sometimes presents itself under strong immobilization or little movement. It is a sub-role that can be quickly switched to the guard repositor sub-role, for example.
BACK TRUNK MOUNTER*(montador do tronco de costas)*	The fighter seeks to mount on the trunk of the opponent who is on their back. This constitutes a different sub-role from the previous one, as the rules state that both situations can be sought in a direct transition from the front mount to the back mount. Again, through the analysis of the fight videos, the internal logic of BJJ indicates that equivalences will only appear within each of these controls and domains, not all of which can be considered as a single sub-role. It is necessary to look at both the scoring rules and the relationship with the opponent (target space: part of the body to be dominated, controlled, crossed, transited, or avoided). This is a scoring sub-role (four points) when: the athlete who is on top and already free of the half-guard sits on the opponent's torso with their back and keeps both knees or one foot and one knee on the ground, facing the opponent's head opponent and with up to one arm of the opponent trapped under their legs, keeping it that way for three seconds (16).
KNEE-ON-BELLY PLACER*(colocador do joelho na barriga)*	The free guard fighter has the clear intention of placing their knee or shin over the opponent's belly, chest, or ribs to dominate them. This is a scoring sub-role (two points) when: the athlete who is on top and free from the guard, places the knee or shin (of the leg closest to the opponent's hip) on the opponent's belly, chest, or ribs, and who must be either standing upright, on their back, or on their side, keeping him/herself stable in this position for three seconds without the opposite knee touching the floor (16).
TRUNK CONTROL ESCAPER*(escapador do controle do tronco)*	The fighter tries not to accept control/immobilization of their torso by the opponent, pushes and moves away from the opponent but does not demonstrate any other tactical intention besides avoiding being immobilized. They can even try to reverse the position from bottom to top in relation to the opponent to escape. This sometimes presents itself under strong immobilization or little movement. After escaping from the immobilization, they can demonstrate a motor behavior to leave that position with a visible intention of restoring their guard; thus, they are already in another sociomotor sub-role.
GUARD RECOVERY*(recuperador de guarda)*	The fighter no longer has the opponent under their guard and tries to have their opponent between their legs again, aiming to have their opponent under their leg guard. They work to embrace at least one of the opponent's legs with their legs, seeking space by pushing, pulling the opponent, evading the hip, or rolling over their shoulder in an attempt to find space to imprison the opponent between their legs again.
BACK-MOUNTED ESCAPER *(escapador da montada de costas)*	The fighter, in an attempt to prevent the opponent from mounting on their trunk from the back, or when already mounted, defends themselves by pushing the opponent's legs, unbalancing them and trying to find space to escape and seek another motor interaction. This sometimes presents itself under strong immobilization or little movement.
KNEE-ON-BELLY ESCAPER*(escapador do joelho na barriga)*	This sub-role specifically seeks to remove the opponent's knee from the fighter's belly, escaping the opponent's domain (pushing the knee, straddling the hip to get the opponent's knee off their belly, etc.).

“*Identifying a new phenomenon and defining it is also acting. It is looking at the land with different eyes. Scientific language is not opposed to technical language: both complement each other. The conquests of physical education will also be those of its language”* (17). Due to the unprecedented nature of the praxiological analysis, all sub-roles were named for the first time. Some names, inevitably, are similar or the same as those spoken in the academies where BJJ is practiced, because they are motor interactions of the practice itself. However, based on the theory of motor action, they gain a specific operational meaning that cannot have a double-meaning. This is what Parlebas (7) conceptualizes as an “operational definition”:

*“… definition with a predominance of the descriptive, enunciated in concrete terms of operations or observable actions, whose objective is to isolate the pertinent identifiable characteristics and susceptible to being subjected to control and, eventually, also measures”* (7).

The sociomotor sub-roles were named considering the dominant motor interaction. Fighters are not obliged to fulfill all the sub-roles during a fight; however, when fighting BJJ they will have their motor acts channeled into some of them according to their interactions with the opponent, their strategies, motor decisions, technical-tactical level, affective and relational motivations, characteristics and physical capacities, as well as by the characteristics of the teaching-learning process of the school/academy. Therefore, the concept of IL is fundamental because it denotes, on the one hand, the presence of a system linked to the ludomotor contract and, on the other hand, it assumes a purpose and a praxis significance of the individual behaviors engendered by this system (7).

In general, considering the descriptions of the BJJ sub-roles, this Brazilian combat sport is characterized by presenting different motor interactions of grappling, dodging, grappling breaks, expectation, guard controls and their breaks, transitions, passes, inversions, domains, and submissions, as well as attempts to escape and defend these interactions. It is worth noting once again that all these interactions take place on the opponent's body at an almost zero guard distance without the possibility of percussive blows such as kicks, punches, elbows, and knees, among others, or the use of instruments such as sticks or swords.

### 3.2. Internal logic of Brazilian jiu-jitsu: the Ludogram of sociomotor sub-roles

**Figure 1:**
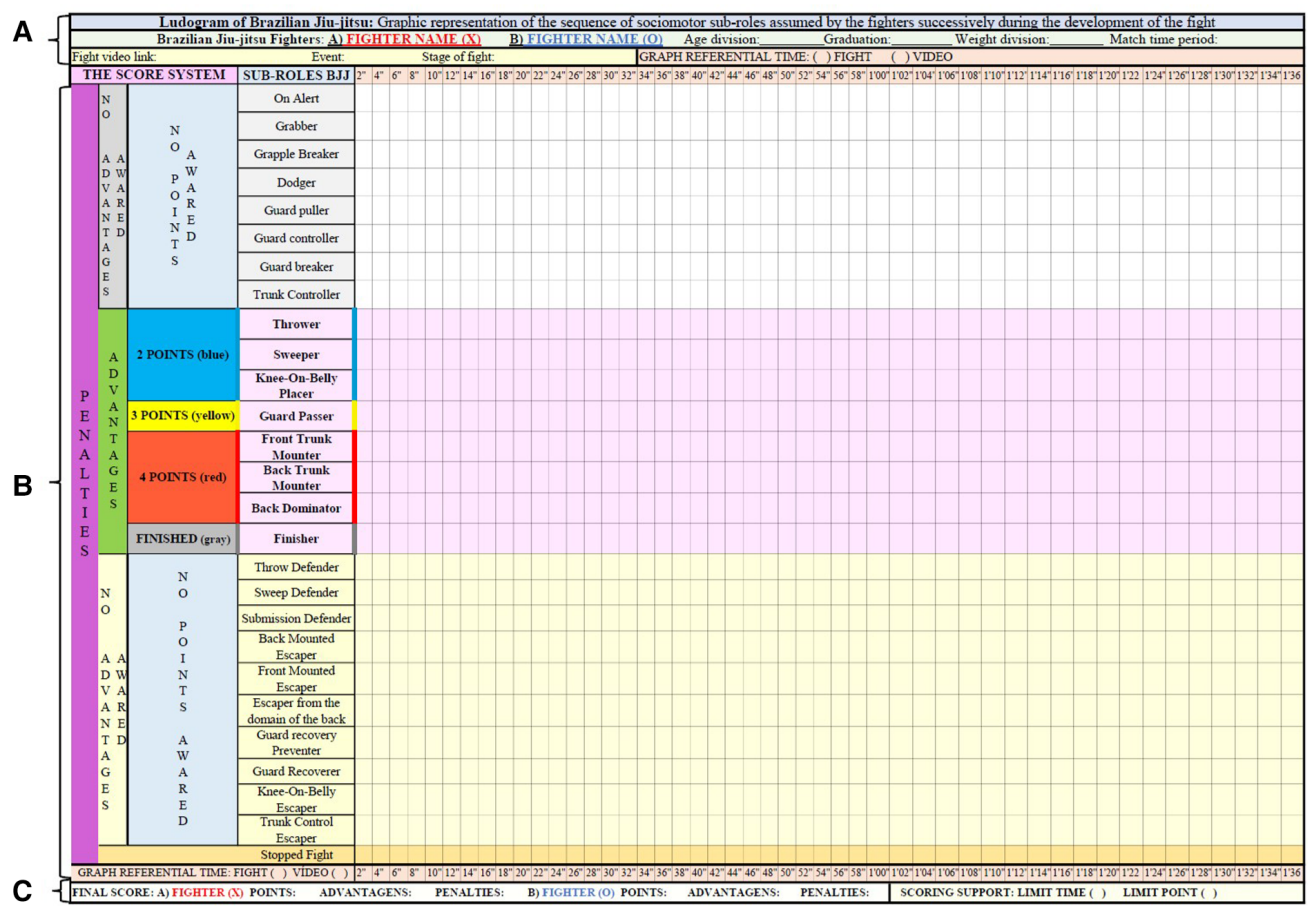
Ludogram of Brazilian jiu-jitsu. (**A**) general information; (**B**) score system, sub-roles, and graphic record; (**C**) final score and score support.

From the 26 identified and described sub-roles, it is possible to build the BJJ Ludogram. The Ludogram consists, according to Parlebas (7), of the “graphic representation of the sequence of sociomotor sub-roles (and eventually the sociomotor roles) assumed by a player, successively, during the development of a sports game”. The author also points out that this valuable instrument will be able to support deeper analyses such as the nature of motor decisions, characteristics of motor behaviors, motor strategy, and the variability of praxic sequences. With this, it is possible to highlight the possibilities that this tool can represent to know and understand the motor conducts of a BJJ fighter. Along with this, this theoretical instrument enables the graphic representation of the sub-roles assumed by the fighter throughout the duration of a fight. Here, the BJJ Ludogram (Figure 1) is presented with an explanatory caption for each of the variables that make up the graph so that teacher-researchers can become familiar with the instrument.

In key (A), there is the general information of the Ludogram: identification of the participating fighters (name, age division, graduation, weight categories, time of the fight, video link of the fight, event, and phase of the fight) and the time of reference for analysis of the itineraries of sub-roles in relation to the time of the fight or the time of the analyzed video.

Key (B) of the Ludogram contains three sections: the score system, sub-roles, and the chart recording the motor itineraries. Regarding the score, it is possible to identify the number of points for each sub-role, punishments, and advantages. The score of the sub-roles is identified by colors: blue sub-roles are worth two points; yellow sub-roles are worth three points; red sub-roles are worth four points; and gray identifies a finalization sub-role. Punishments can be signaled in any of the sub-roles, while the accounting of advantages refers to the scoring sub-roles.

In the second section of key (B), the 26 BJJ sub-roles are presented, which were categorized into three sub-role bands and one band (Stopped Fight) to identify the record of fight interruptions during the confrontation. Each sub-role band is detailed below.

- **White band, referring to non-scoring sub-roles**: on alert, grabber, grapple breaker, dodger, guard puller, guard controller, guard breaker, and trunk controller.
- **Light pink band, referring to the scoring sub-roles**: thrower, sweeper, and knee-on-belly placer (two points); guard passer (three points); and front trunk mounter, back trunk mounter, and back dominator (four points).
- **Light yellow band, the sub-roles of escapes, defenses, throws, and impediment**: throw defender, sweep defender, submission defender, back-mounted escaper, front-mounted escaper, escaper from the domain of the back, guard recovery preventer, guard recoverer, knee-on-belly escaper, and trunk control escaper.
- **Light orange band, indicates the stopped fight moments throughout the combat**: interruptions made by the arbitration (exit from the fight area, adjustment of the fighters' kimono, etc).

The third section of key (B) consists of marking the itineraries of the sub-roles assumed by the fighters in relation to the time of the fight or the analyzed video. This space on the chart is intended to record the trajectory of the sub-roles successively adopted by the fighters. To mark and differentiate the itineraries taken by the fighters in relation to the time of fight or video, one fighter is identified with the caption (X) in red and the other with (O) in blue. Due to the speed of BJJ sub-role changes, it is suggested that each quadrant on the horizontal axis indicate the time in seconds (between 1 and 2 s) or as needed.

**Figure 2:**
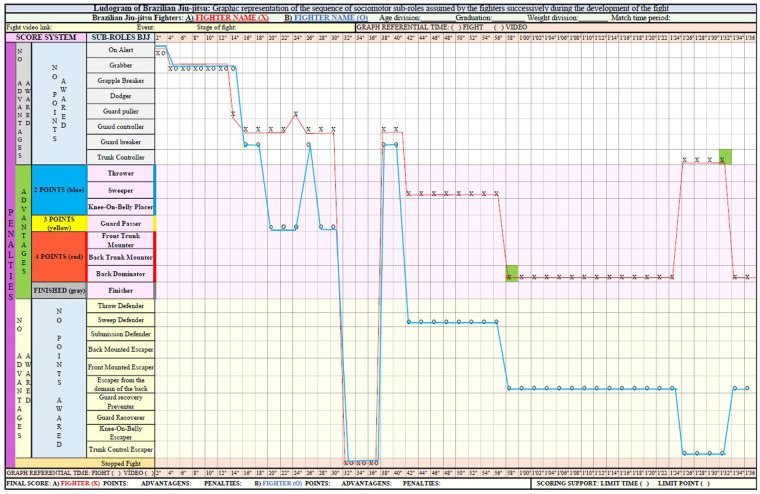
Example of a Ludogram recording of a BJJ fight (part 1).

**Figure 3:**
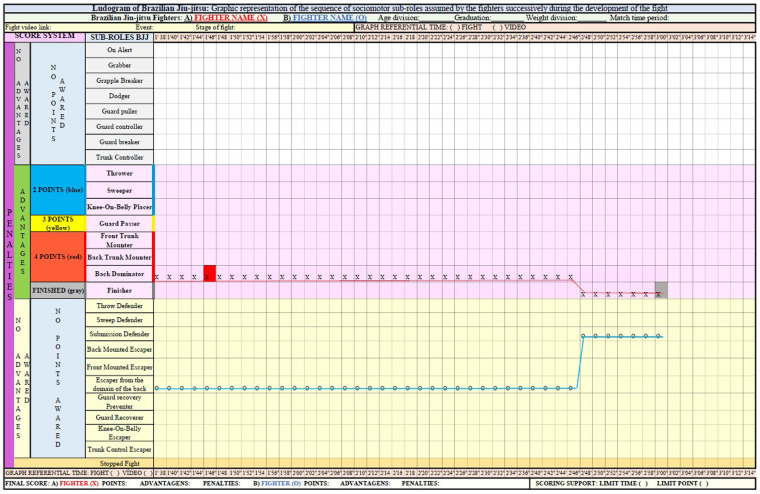
Example of a Ludogram recording of a BJJ fight (part 2).

At the bottom of the graph, key (C), the final score of each fighter is presented in relation to the score, advantages, and penalties, as well as the score support that will indicate whether the fight was finalized by time limit or score limit. After these initial highlights, Figures 2, 3 show an example of a Ludogram with the graphic record of the fighters' itineraries.

These two figures represent the records of the itineraries of the sub-roles of the two fighters identified and differentiated by the legend X and O of a fight from the beginning to the end. In addition to the caption in each space of time, it is necessary to draw a line that expresses the path taken by the fighters, allowing a better visualization of the dynamics of exchanges of the sub-roles during the combat.

In addition to recording the itineraries undertaken by the fighters, it is possible to express in the Ludogram the advantages, penalties, submissions, scores, and their values along the graph that are identified by colors. This information is marked in the quadrant with the corresponding color in relation to the fight time in which it is obtained. Therefore, it is necessary for the observer to enter the corresponding color of the score, penalty, or advantage in the quadrant in relation to the fight time or video for each fighter. At the end, the researcher/teacher will have, in addition to the assumed sociomotor sub-roles, the record of successes and failures regarding the scoring system. With that, the Ludogram also expresses the score interactions achieved by BJJ fighters.

## 4. Conclusion

The results of this research regarding the identification and description of the BJJ sub-roles corroborate and expand the analyses by Schmidt and Ribas (5), who pointed out that this is a body combat sport practiced in a stable environment with oppositional sociomotor interaction; a duel between two individuals with a target-space objective of the motor interactions in the opponent's body with motor interactions pertinent to practices classified in the almost zero guard distance (gloveless combatants, with permanent contact between the fighters that authorize and codify the combat on the ground, and with one of the objectives being to knock down the opponent).

The identification and description of the BJJ sub-roles revealed a complex web of possibilities imposed on the participating fighters by their internal logic. It qualifies BJJ as an oppositional socio-motor practice that requires from its fighters incessant decision-making and modifications in their motor strategies during the duel.

The 26 identified and described sub-roles of BJJ indicate the richness of choices and possible paths to be followed by fighters within this itinerary of motor interaction. They are: on alert, grabber, grapple breaker, dodger, guard puller, guard controller, guard breaker, and trunk controller; thrower, sweeper, and knee-on-belly placer; guard passer, front trunk mounter, back trunk mounter, and back dominator; and throw defender, sweep defender, submission defender, back-mounted escaper, front-mounted escaper, escaper from the domain of the back, guard recovery preventer, guard recoverer, knee-on-belly escaper, and trunk control escaper. These different BJJ sub-roles described in this research highlight the importance of the concept of praxis communication, specifically, motor counter-communication, since many of the dynamics between a fighter's sub-roles refer to the choices that the opponent indicates for the motor dialogue.

If on the one hand there are the less desired sub-roles, on the other hand, there are the preferred itineraries, those with greater control and, if that weren't enough, some of them modify the score. Of the 26 sub-roles revealed, can modify the score of this combat sport.

Respecting the specific scoring rules, when successfully assumed by the participating fighter, the sub-roles of thrower, sweeper, knee-on-belly placer, guard passer, front trunk mounter, back trunk mounter, and back dominator add up to points on their score over the regular time of the fight, but they are not able to end the fight by maximum score. This is only possible when the fighter is successful in the finisher sub-role, which ends the fight before the maximum time limit set, usually due to the opponent's withdrawal and/or submission.

The characteristic of BJJ's internal logic referring to the score shows that this Brazilian combat sport significantly altered its motor objectives. Before, if the objective of the fight was self-defense and submission of the opponent, now, with the creation of the scoring system, the fighters can also guide their motor decisions towards score counter-communication interactions.

Each sub-role safeguards the objective characteristics of the interaction logic, but they will be assumed by different subjects of the action who will express their personality through each one of them. Are affective, relational, cognitive, and organic issues similarly activated in the different BJJ sub-roles? Is overcoming oneself in front of the opponent exactly the same in the sub-roles that allow scoring in relation to those that do not? Would relativizing defeat and victory also be the same in view of the different sub-roles that a fighter assumed during a fight? Will losing and winning a fight through maximum score (success in the sub-role of submission, failure in the sub-role defender of submission) reveal the same activations on the motor behaviors of those fighters who won or were won by score?

Recognizing the motor behavior of BJJ fighters is looking beyond the techniques or movements and trying to recognize the subject in its entirety during the motor actions in order to serve to establish new pedagogical objectives. It reinforces the importance of the need for experience and experience that each of these dynamics and minimum interactions of BJJ can request from participating fighters. It is emphasized that the motor behavior and its competences (cognitive, affective, relational, and organic) must necessarily be observed during the motor practice as a behavioral procedural knowledge and not as a declarative knowledge. It is in this sense that the Ludogram stands out as a theoretical-scientific instrument that can support the objective understanding of this behavioral procedural knowledge based on the internal logic-motor behavior relationship.

The BJJ Ludogram enables future praxeological analyses of the sub-roles and motor behaviors of any subject who wants to assume the sociomotor role of a BJJ fighter according to the rules of this Brazilian combat sport. The characteristic variables of the players, age, sex, fighter's belt Üategory, capacity for initiative, and technical-tactical levels, among others, will be able to describe and aid understanding of different groups of fighters in terms of their motor behavior and motor conduct. It is important to emphasize that the instrument should be validated in future studies.

BJJ, as a practice with opposition interaction, requires from participating fighters incessant activations on aspects related to socio-motor intelligence, such as the need for socio-motor empathy, motor strategy, pre-acting, developing the capacity to make motor decisions, the ability to anticipate anticipations, to recognize the affective, cognitive, relational, and organic loads activated during the fight, and to develop their motor behavior.

Within the scope of didactic-pedagogical implications, the teacher/coach in possession of this new knowledge related to this motor practice and, inserted in a concrete reality, will be able to evaluate and plan the pedagogical interventions, bringing students/athletes closer to significant experiences of this motor practice when developing the pedagogical objectives coherently with the possibilities that this inseparable internal logic-motor behavior relationship can provide.

Thinking through the MP and the IL relationship and the concept of motor behavior means abandoning a mechanistic conception of physical education to introduce possibilities for a systemic, contextualized, and optimized physical education in its place. At the same time, it is necessary to dialectically articulate the internal logic with the external logic to enable a critical understanding of the social context of the social context in pedagogical practice. If there is an internal logic, it means understanding that there is also an external logic. From this relationship, it is understood that the starting point for this problematization in the scope of pedagogical practices, which work with sports games, consists of understanding that the very motor behavior of the subject who plays, fights, and participates in the praxeological system already presents elements of external logic.

The activations of the motor behavior of the subject of the action provoked by the internal logic of a sports game can be an effective way of bringing to light the traits of the society in which this historical subject is inserted, when considering the different dimensions of the motor behavior (organic, relational, cognitive, affective, social, etc.). That is, at the same time that it is inserted in an IL of a sports game, it also contains registered in its motor behavior aspects of the external logic in which it is also inserted. This question can be expanded even more if the concepts proposed by Pierre Parlebas of ethnomotricity, habitus, and playful contract with internal logic are articulated, a step that is only possible with the development of praxeological science.

The knowledge generated by the description of the internal logic of BJJ, specifically the identification and description of the sociomotor sub-roles and the production of the Ludogram, indicate new possibilities for scientific studies, among them: to infer the didactic-pedagogical implications of these results in pedagogical practices; to understand/signify the motor behaviors of the participating fighters based on the praxeological system and subject of action in BJJ; and to produce new analyses based on the Ludogram about participating fighters (decision making, fight strategies, etc.) in the different contexts in which BJJ is inserted.

## Data Availability

The original contributions presented in the study are included in the article, further inquiries can be directed to the corresponding author.
